# A Provably Secure IBE Transformation Model for PKC Using Conformable Chebyshev Chaotic Maps under Human-Centered IoT Environments

**DOI:** 10.3390/s21217227

**Published:** 2021-10-30

**Authors:** Chandrashekhar Meshram, Agbotiname Lucky Imoize, Amer Aljaedi, Adel R. Alharbi, Sajjad Shaukat Jamal, Sharad Kumar Barve

**Affiliations:** 1 Department of Post Graduate Studies and Research in Mathematics, Jaywanti Haksar Govt. Post-Graduation College, College of Chhindwara University, Betul 460001, India; cs_meshram@rediffmail.com; 2 Department of Electrical and Electronics Engineering, Faculty of Engineering, University of Lagos, Akoka, Lagos 100213, Nigeria; 3 Department of Electrical Engineering and Information Technology, Institute of Digital Communication, Ruhr University, 44801 Bochum, Germany; 4 College of Computing and Information Technology, University of Tabuk, Tabuk 71491, Saudi Arabia; aaljaedi@ut.edu.sa (A.A.); aalharbi@ut.edu.sa (A.R.A.); 5 Department of Mathematics, College of Science, King Khalid University, Abha 61413, Saudi Arabia; shussain@kku.edu.sa; 6 Water Resources and Applied Mathematics Research Lab, Nagpur 440027, India; drshardbarve@rediffmail.com

**Keywords:** public key cryptography, identity-based encryption schemes, Chebyshev polynomial, conformable Chebyshev chaotic maps, human-centered Internet of Things

## Abstract

The place of public key cryptography (PKC) in guaranteeing the security of wireless networks under human-centered IoT environments cannot be overemphasized. PKC uses the idea of paired keys that are mathematically dependent but independent in practice. In PKC, each communicating party needs the public key and the authorized digital certificate of the other party to achieve encryption and decryption. In this circumstance, a directory is required to store the public keys of the participating parties. However, the design of such a directory can be cost-prohibitive and time-consuming. Recently, identity-based encryption (IBE) schemes have been introduced to address the vast limitations of PKC schemes. In a typical IBE system, a third-party server can distribute the public credentials to all parties involved in the system. Thus, the private key can be harvested from the arbitrary public key. As a result, the sender could use the public key of the receiver to encrypt the message, and the receiver could use the extracted private key to decrypt the message. In order to improve systems security, new IBE schemes are solely desired. However, the complexity and cost of designing an entirely new IBE technique remain. In order to address this problem, this paper presents a provably secure IBE transformation model for PKC using conformable Chebyshev chaotic maps under the human-centered IoT environment. In particular, we offer a robust and secure IBE transformation model and provide extensive performance analysis and security proofs of the model. Finally, we demonstrate the superiority of the proposed IBE transformation model over the existing IBE schemes. Overall, results indicate that the proposed scheme posed excellent security capabilities compared to the preliminary IBE-based schemes.

## 1. Introduction

Human-centered Internet of Things (IoT) enables seamless processing of electronic transactions, healthcare information systems, efficient operation of intelligent devices, and more [1]. However, the design of modern human-centered IoTs poses several opportunities to be harnessed and several challenges that need to be addressed appropriately. Currently, several massive devices are incorporated into human-centric IoT systems, thereby enabling the exchange of user information over public communication channels [2]. However, the problem of preserving user confidentiality and privacy over such channels remains. Thus, there is a need for an efficient and reliable security mechanism, based on chaotic frameworks, to guarantee secure information exchange via the deployment of lightweight security schemes for application in human-centered IoT environments.

Additionally, the IoT has brought dramatic changes in modern society, leading to significant improvements in our daily interactions with the natural environment [3,4,5]. In the human-centered IoT environment, billions of smart devices are interconnected to communicate with each other wirelessly [6]. In an active wireless network, smart devices orchestrate user data collection, analysis, and user data sharing in real-time [7]. Due to the complexity of the wireless network infrastructure, its vulnerability to several malicious and adversarial attacks becomes an issue that requires urgent attention. In order to address this problem, there is a need to provide the requisite authentication and confidentiality based conformable Chebyshev chaotic maps (CCCM) [8] for sensitive user data before transmission over public wireless channels, which is the motivation for the current study.

In recent years, research in chaotic maps and their applications within cryptography has acquired significant attention. Chaotic frameworks display features that appear to be fundamentally analogous to those needed by certain cryptographic primitives. In practice, Chebyshev chaotic maps play significant roles in increasing the security of cryptographic schemes and decreasing the communication overhead compared to pairing, elliptic curve, etc. For instance, the work of [8] is based on extended chaotic maps, whereas our proposed work is based on conformable Chebyshev chaotic maps.

Chebyshev polynomial and conformable calculus have been used in the design of the proposed IBE scheme. Conformable calculus plays a significant role in increasing the security of cryptographic schemes compared to cryptographic schemes based on chaotic maps, enabling the selection of arbitrary rational numbers 𝛾∈[0, 1]. Here, the foe cannot calculate the arbitrary rational number via guessing the security of the system. Additionally, the proposed scheme requires minimum communication cost compared to the scheme reported in [8]. 

### 1.1. Contribution

This paper presents a provably secure IBE transformation model under the human-centered IoT contexts. The model can convert a conformable Chebyshev chaotic map-based PKC into secure IBE utilizing a conformable Chebyshev chaotic map without requiring the creation of a new framework. In particular, our novel approach includes a key generation stage (KGS) with extraordinarily low processing computation complexity. Furthermore, the original conformable Chebyshev chaotic map in the PKC does not need to be adjusted using our new model. By converting a conformable Chebyshev chaotic map-based PKC into an IBE using a conformable Chebyshev chaotic map, our new model provides the same level of suitability and client-friendliness as the original conformable Chebyshev chaotic map-based PKC. In this case, individual users can choose their names or network addresses as their identifiers. As a result, public-key confirmation seems quite natural and straightforward.

Interestingly, there is no need for an extensive public key database under the proposed architecture. Furthermore, we present a reductionist security investigation against the selective identity adaptive chosen ciphertext attack (IND-sID-CCA) in the ROM. In particular, the key contributions of the paper are outlined as follows:

■ We provide a provably secure IBE transformation model under the human-centered IoT contexts comprising a KGS with extremely low processing computation complexity;
■ We show that the presented transformation procedure is accomplished by interpreting a well-designed and secure conformable Chebyshev chaotic map-based scheme into an equally robust ID-based cryptosystem under human-centered IoT environments;
■ We demonstrate that our new model provides the same level of suitability and client-friendliness compared to the original conformable Chebyshev chaotic map-based PKC;
■ We show that there is no need for an extensive public key database under the new architecture;
■ We test the proposed ID-based system against IND-sID-CCA in the ROM by employing the reductionist method.

### 1.2. Paper Organization

The following is how the rest of the article is structured. The related works are summarized in Section 2. In Section 3, some background materials on conformable Chebyshev chaotic maps are presented. In Section 4, we describe our IBE transformation model for PKC in human-centered IoT contexts. An example of how the suggested framework operates is also provided to help demonstrate its viability. After that, in Section 5, the security analysis and prospects of the new IBE transformation model are examined. The projected model is compared to several other related models in terms of efficiency and performance. Finally, in Section 6, the conclusion to the paper is drawn.

## 2. Related Works

In the existing literature, several public-key cryptographic (PKC) and signature-based schemes have been proposed to provide seamless and secure communications of critical user data [9]. However, smart devices require lightweight operations to guarantee extensive security requirements. In recent years, digital signatures have been proposed to guarantee the authenticity and confidentiality of user information. However, the problem of building a robust public-key database to generate millions of public-key certificates remains uncharacterized. Consequently, several schemes have been projected to address this problem in IoT-based systems. 

Real-world security and IoT-based solutions are susceptible to various attacks that pose severe security and privacy issues. Recently, intrusion detection systems (IDSs) have been deployed in [3] to address these issues. In particular, the work proposed software-defined IDS-based distributed cloud architecture to secure an IoT platform. The results showed impressive security against tested attacks. The work in [10] suggested a password-enabled lightweight authentication protocol, smart card, and biometric identification. The scheme achieved mutual authentication among users with increased nonrepudiation. 

In [4], multi-level decomposition feedback provided an evaluation model for user information security. Four novel indicators were used to supervise the performance of the evaluation model better, and simulation results show the suitability of the proposed model for the security of IoT systems. Similarly, a new scheme based on secure architecture for energy-efficient IoT in edge infrastructure was proposed [11]. Blockchain-enabled distributed network is used at the fog layer for security and privacy. Performance evaluation of the scheme showed superior features to the existing schemes. In [5], a multi-level key exchange and encryption protocol for IoT was proposed. The work presents a secret key generation technique and a new authentication protocol for enhanced security.

In 1984, Shamir [12] introduced an identity-based cryptosystem (IBC) that needs no certificates to secure critical user information over public channels. The Cocks IBE scheme [13], which encrypts the plaintext into ciphertext, is another encryption algorithm worth mentioning in this paper. However, the performance of this algorithm depends on the computational processing time of integer factorization, which is cumbersome to resolve in practice. 

Furthermore, the Boneh–Boyen IBE scheme [14] is a special IBE scheme used to encrypt the identity of the users in a well-defined security system. Additionally, Boneh and Franklin [15,16] presented a provably secure and implementable IBC scheme that uses pairing. However, the scheme has a high computation processing time, which poses a significant limitation. Recently, Sakai–Kasahara [17] reported a bilinear pairing-based IBE scheme faster than the Boneh and Franklin scheme. It is worth mentioning that the Sakai–Kasahara scheme shows superior security features to the Boneh and Franklin scheme as it does not use modular exponentiation. However, the scheme possesses computationally intensive characteristics, which limits its usefulness in lightweight IoT-centered environments. 

It is immensely gratifying to note that the emergence of the pairing-based IBC scheme has opened a new frontier of research in the public key cryptography domain [18,19,20,21,22,23,24]. Therefore, the current undertaking aimed at a provably secure IBE transformation model for PKC using conformable Chebyshev chaotic maps under human-centered IoT environments is not out of place.

In human-centered IoT environments, application-specific signatures have been advanced [25,26,27,28,29,30,31,32]. In particular, the work in [25] proposed a signature scheme that does not use the random oracle. In [26], optimized security schemes that enable message signature independent of online computation were presented. Furthermore, Guo et al. [27] extended the scheme in [26] to accommodate online computational leakage resilience. In work due to Yao and Zhao [28], signature schemes were designed specially for low-power applications. Additionally, signature schemes have been reported for wireless sensor networks [29,30]. Also, Zheng et al. [31] have reported signatures that use lattice. Furthermore, Addobea et al. [32] put up a certificateless signature scheme for medical devices. However, most of the signature schemes are based on pairing, and such IBS constructions require complex pairing operations in groups, which poses substantial computational costs. 

In recent times, IoT smart devices have been integrated with sensors to facilitate user data sharing in public wireless channels. However, sensors are designed with limited storage and computing resources. Therefore, lightweight and energy-saving schemes are highly coveted to guarantee the authentication and confidentiality of IoT-based systems. Toward this end, Even et al. [33] introduced a special-purpose signature, which finds practical applications in lightweight devices. Liu et al. [21] suggested an efficient, provably secure IBS technique that uses multi-time usage of offline storage. In [34], an IBS scheme derived from Hohenberger’s RSA signature [35] was examined, highlighting its flaws. Similarly, the concept of certificateless cryptography [9] was employed by Liu et al. [36] to present an identity-based signature scheme that does not require pairing. 

Recently, Meshram et al. [37] presented a provably secure scheme using extended chaotic maps. Guo et al. [38] also provided an extended signature technique and applied it to IBE. In Guo et al.’s scheme, lightweight computations are performed in the online encryption phase, while the offline encryption phase carries out computationally intensive tasks. In related work, Liu and Zhou [39] reported an efficient IBE scheme with short ciphertexts and claimed its superiority over the preliminary schemes. 

The encryption and decryption phases of the scheme demonstrated by Liu and Zhou have significant improvements in computational costs compared to the scheme reported in [30]. Additionally, an identity-based key encapsulation scheme with security against chosen-ciphertext attacks was presented in [40]. Selvi et al. [41] identified some flaws in the scheme proposed in [39] and improved the scheme to achieve CCA security. Towards this end, an ordinary IBE scheme was converted to an online/offline IBE scheme by Lai et al. [42]. Similarly, the authors in [43] reported an IBE scheme for lightweight devices, and the work in [44] extended the technique of online/offline to attribute-based encryption. 

In recent times, the work in [45] presented a new one-dimensional chaotic map based on a simple iterative mathematical equation. Intel I7-7700HQ processor with 16 GB RAM powers the experimentation environment. The proposed map shows a simple structure, a high chaotic behaviour, an infinite chaotic range, and is suitable for the design of chaos-based cryptographic systems. The scheme also offers a better security level and a higher encryption speed. In recent times, chaotic techniques have been found in diverse fields, like cryptosystems and image encryption. In particular, the work in [46] presents a novel method for digital image encryption. The simulation and theoretical analysis of the scheme indicate that this scheme is suitable for actual image encryption.

Recently, a robust elliptic curve–based image encryption and authentication model for grayscale and colour images have been presented [47]. The model uses the secure elliptic curve Diffie–Hellman key exchange to compute a shared session key with the enhanced ElGamal encoding scheme. The model shows low computational costs with minimized point multiplication operations and resilience against chosen-plaintext, known-plaintext, and occlusion attacks. Similarly, a new image encryption scheme based on chaotic hybrid maps is presented in [48]. The scheme employs both the confusion phase to scramble the location of pixels and the diffusion phase for consecutively changing the content of pixels. The scheme shows more comprehensive chaotic behaviour with excellent encryption and decryption processing time.

The work in [49] proposes a new fractional one-dimensional chaotic map with a sizeable chaotic space. The proposed scheme has a simple structure and high chaotic characteristics. The proposed map was also used in the design of a novel real-time image encryption scheme. Simulation tests and experimentation prove that the method has high performance and is highly efficient. A novel video watermarking scheme using a two-dimensional complex chaotic map is presented in [50]. The simulation results showed that the scheme has good visual quality using standard criteria. The scheme was also tested using geometric and non-geometric attacks and offered robust security against all tested attacks.

Given the preceding literature, the IBS signcryption, capable of encryption and signature, has been well investigated [21,51,52]. Although IBE schemes have been studied for several decades, to the authors’ best knowledge, there is no provably secure IBE transformation model for PKC using conformable Chebyshev chaotic maps, especially under human-centered IoT environments. However, encryption and decryption or signature and verification processes require large real numbers, which incur substantial computational overhead. In order to address this problem, this paper proposes a provably secure IBE transformation model for public-key cryptography leveraging conformable Chebyshev chaotic constructions. In particular, the model is designed to generate security credentials for verification and signature processes at a minimal computational cost. It is immensely gratifying that the proposed scheme does not use extensive operations to process encryption and decryption. Additionally, the scheme is protected under adaptive chosen message attack in the random oracle model (ROM). A brief comparison of related works with our proposed work is given in Table 1.

## 3. Background and Materials

This segment reviews the various underlying concepts relating to the work, before delving into the current investigation on the IBE transformation model for PKC using conformable Chebyshev chaotic maps under the human-centered IoT environments. First, a short-lived Chebyshev chaotic map implementation is presented. This is followed by a Chebyshev polynomial, conformable Chebyshev chaotic maps, using the minimal method. A list of symbols used in the paper is given in Table 2.

### 3.1. Chebyshev Chaotic Polynomials

Chebyshev sequential polynomials (CSP) operatory is examined (see [53]). CSP ${Ƭ}_{ᶇ}\left(ʑ\right)$ is a ᶇ-degree polynomial in the variation. Let ʑ∈[−1, 1] be the arrangement, and ᶇ be an integer. CSP reported the following in general: ϯ

$${Ƭ}_{ᶇ}\left(ʑ\right)=\cos\left(ᶇ\times \cos^{-1}\left(ʑ\right)\right),\;{Ƭ}_{0}\left(ʑ\right)=1,\;{Ƭ}_{1}\left(ʑ\right)=ʑ,\;{Ƭ}_{ᶇ}\left(ʑ\right)=2ʑ{Ƭ}_{ᶇ-1}\left(ʑ\right)-{Ƭ}_{ᶇ-2}\left(ʑ\right);\;ᶇ\geq 2$$

Under these circumstances, the functional $\cos^{-1}\left(ʑ\right)\;$ and  cos(ʑ) are represented as $\cos^{-1}:\;\left[-1,\;1\right]\to \left[0,\;\pi \right]\;$ and  cos: Ɍ→[−1, 1]. 

The chaotic and semi-group features of CSP are fundamental [37,54,55,56,57].

The chaotic feature: The CSP map is defined as ${Ƭ}_{ᶇ}:\;\left[-1,\;1\right]\to \;\left[-1,\;1\right]\;$with degree  ᶇ > 1, is a chaotic map accompanying with the (invariant density) functional $\;f^{*}\left(ʑ\right)=\frac{1}{\left(\pi \sqrt{1-{ʑ}^{2}}\right)}$ for the positive Lyapunov exponent  λ=lnᶇ>0.

(1) Semi-group feature: A semi-possession group must meet the following criteria:

$${Ƭ}_{\ell }\left({Ƭ}_{𝓌}\left(ʑ\right)\right)=\cos\left(\ell \cos^{-1}\left(\cos\left(𝓌\cos^{-1}\left(ʑ\right)\right)\right)\right)\;=\cos\left(\ell 𝒸\cos^{-1}\left(ʑ\right)\right)\;={Ƭ}_{𝓌\ell }\left(ʑ\right)\;={Ƭ}_{𝓌}\left({Ƭ}_{\ell }\;\left(ʑ\right)\right),$$

where  ʑ∈[−1, 1] and ℓ and 𝓌  are positive integers.

Zhang [58] demonstrated that the semi-group assets preserve the (−∞,+∞) interval, which may be used to improve the property as tracks:

$${Ƭ}_{ᶇ}\left(ʑ\right)=2ʑ{Ƭ}_{ᶇ-1}\left(ʑ\right)-{Ƭ}_{ᶇ-2}\left(ʑ\right);\;n\geq 2$$

where  ʑ∈(−∞,+∞) and $q_{1}$ is a large and safe prime. As a result, the property is:

$${Ƭ}_{\ell }\;\left({Ƭ}_{𝓌}\left(ʑ\right)\right)\left(modq_{1}\right)={Ƭ}_{𝓌\ell }\left(ʑ\right)\left(modq_{1}\right)={Ƭ}_{𝓌}\left({Ƭ}_{\ell }\;\left(ʑ\right)\right)\left(modq_{1}\right)$$

Furthermore, the semi-group property is preserved. It is worth mentioning that extended Chebyshev polynomials commute in the presence of confirmation.

For Chebyshev polynomials (CP), there are two assessments that evaluate handling in polynomial time:

(1) Given two ʑ and  𝓋, the objective of the discrete log (DL) is to invent an integer ℓ with the ultimate aim ${Ƭ}_{\ell }\left(ʑ\right)=𝓋$.
(2) The goal of the Diffie–Hellman problem (DHP) is to calculate the ${Ƭ}_{\ell 𝓌}\left(ʑ\right)$ element using three elements: ʑ, ${Ƭ}_{\ell }\left(ʑ\right)$, and ${Ƭ}_{𝓌}\left(ʑ\right)$.

### 3.2. Conformable Chebyshev Chaotic Maps (CCCM) 

The conformable calculus (CC) was previously referred to as the conformable fractional calculus (CFC) [59]. However, it places a strain on the established fractional calculus properties (derivatives of non-integer power). In essence, CC is in charge of future planning. The proposed work is founded on conformable Chebyshev chaotic maps. This implies that the development of the proposed IBE scheme depends on Chebyshev polynomial and conformable calculus. Notably, conformable calculus plays a significant role in enhancing the security of cryptographic schemes, compared to cryptographic schemes based on the chaotic map, due to the selection of arbitrary rational numbers 𝛾∈[0, 1]. That being said, the foe cannot calculate the arbitrary rational number by guessing the security of the proposed IBE scheme.

Assume 𝓎 is a fractional (arbitrary) number in the range of 0 to 1. If, and only if, $\delta^{0}$ is the self-operator and $\delta^{1}$ is the typical difference operational, the operator 𝓎 is conformable differential. $\delta^{𝓎}$ is unambiguously conformable for differentiable utility if, and only if, ξ = ξ $\left({𝓍}_{1}\right)$.

$$\delta^{0}\;\xi \left({𝓍}_{1}\right)=\xi \left({𝓍}_{1}\right),{\;\delta }^{1}\xi \left({𝓍}_{1}\right)=\xi^{\prime }\left({𝓍}_{1}\right).$$

To explain the performance of a proportional-differentiation controller that adheres to the error function, Anderson et al. [59] suggested a novel formulation of CC derived from control theory. The structure of the instruction is as follows.

**Definition 1.**  *CC has in the following documentation if*𝓊*ϵ [0, 1] is true*.

$$\delta^{u}\xi \left({𝓍}_{1}\right)={ϒ}_{1}\left(𝓎,{𝓍}_{1}\right)\xi \left({𝓍}_{1}\right)+{ϒ}_{0}\;\left(𝓎,{𝓍}_{1}\right)\xi^{\prime }\left({𝓍}_{1}\right),$$

where the ${ϒ}_{1}$ and ${ϒ}_{0}$ functions reach the limits

$$\underset{𝓎\;\to 0}{\lim}{ϒ}_{1}\left(𝓎,{𝓍}_{1}\right)=1,\underset{𝓎\;\to 1}{\;\lim}{ϒ}_{1}\left(𝓎,{𝓍}_{1}\right)=0,\;\underset{𝓎\to 0}{\lim}{ϒ}_{0}\left(𝓎,{𝓍}_{1}\right)=0,\underset{𝓎\;\to 1}{\lim}{ϒ}_{0}\left(𝓎,{𝓍}_{1}\right)=1.$$

To obtain the overhead description, we shall deliberate $\;{ϒ}_{1}\left(𝓎,{𝓍}_{1}\right)=\left(1-𝓎\right){𝓍}_{1}{}^{𝓎}$ and ${ϒ}_{0}\left(𝓎,{𝓍}_{1}\right)=𝓎{𝓍}_{1}{}^{1-𝓎}\;$, or ${ϒ}_{1}\left(𝓎,{𝓍}_{1}\right)=\frac{\left(1-𝓎\right)}{\Gamma \left(1+𝓎\right)}\;\mathrm{and}\;{ϒ}_{0}\left(𝓎,{𝓍}_{1}\right)=\frac{𝓎}{\Gamma \left(1+𝓎\right)}\;$ where $\delta^{𝓊}\xi \left({𝓍}_{1}\right)$ is the name of the $\xi \left({𝓍}_{1}\right)\;$function’s conformable differential operator. As a result, the function’s fractional tuning connections with its derivative, ${ϒ}_{1},{ϒ}_{0}$, are always reliable.

The resulting structure is obtained by using the concept of CC to express the polynomial ${Ʈ}_{ᶇ}\left({𝓍}_{1}\right)$:

Since ${Ʈ}_{ᶇ}^{’}\left({𝓍}_{1}\right)=2ᶇ\;{Ʈ}_{ᶇ-1}\left({𝓍}_{1}\right)$, then $\delta^{𝓊}{Ʈ}_{ᶇ}\left({𝓍}_{1}\right)$ has the subsequent formal relationship (1)

(1) $${Ʈ}_{ᶇ}^{𝓎}\left({𝓍}_{1}\right):={\;\delta }^{𝓎}{Ʈ}_{ᶇ}\left({𝓍}_{1}\right)={ϒ}_{1}\left(𝓎,{𝓍}_{1}\right){Ʈ}_{ᶇ}\left({𝓍}_{1}\right)+{ϒ}_{0}\;\left(𝓎,{𝓍}_{1}\right){Ʈ}_{ᶇ}^{’}\left({𝓍}_{1}\right)\;$$

**Figure 1:**
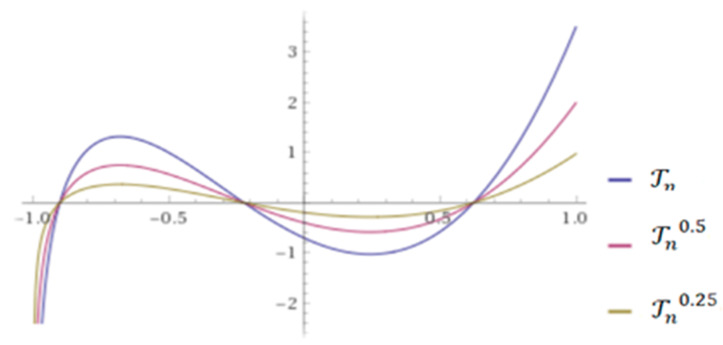
CCP for different values of 𝓎=0.25, 0.5, 1 with ${ϒ}_{1}\left(𝓎,{𝓍}_{1}\right)=\frac{\left(1-𝓎\right)}{\Gamma \left(1+𝓎\right)}\;\mathrm{and}\;{ϒ}_{0}\left(𝓎,{𝓍}_{1}\right)=\frac{𝓎}{\Gamma \left(1+𝓎\right)}$ .

The Formula (1) can be substituted with (2)

(2) $${Ʈ}_{ᶇ}^{𝓎}\left({𝓍}_{1}\right)={ϒ}_{1}\left(𝓎,{𝓍}_{1}\right){Ʈ}_{ᶇ}\left({𝓍}_{1}\right)+2ᶇ\;{ϒ}_{0}\;\left(𝓎,{𝓍}_{1}\right)*\;\omega \left({𝓍}_{1}\right){Ʈ}_{ᶇ-1}\left({𝓍}_{1}\right)\;,\;$$

where $\omega \left({𝓍}_{1}\right)=1+2{𝓍}_{1}+\left(4{𝓍}_{1}{}^{2}-1\right)+\ldots +\left(ᶇ-1\right)$-times. Equation (2) defines the Conformable Chebyshev Polynomials (CCP) and a few numerical examples of CCP are shown in Figure 1 [60].

Properties of CCCM: The following are two intriguing characteristics of the CCCM:

**Definition** **2.**  *(Chaotic properties of CCCM). Under the chaotic property, the CCCM satisfies recurrent relations [8], i.e.,*

$${Ʈ}_{ᶇ}^{𝓎}\left({𝓍}_{1}\right)=[2{𝓍}_{1}\;\eta_{1}\left(\alpha ,{𝓍}_{1}\right)+2ᶇ\;\eta_{0}\;\left(𝓎,{𝓍}_{1}\right)*\;\omega \left({𝓍}_{1}\right)]{Ʈ}_{ᶇ-1}\left({𝓍}_{1}\right)-\eta_{1}\;\left(𝓎,{𝓍}_{1}\right){Ʈ}_{ᶇ-2}\left({𝓍}_{1}\right)\;.$$

**Definition 3.**  *(Semi-group properties of CCCM). The semi-group properties look for CCCMs located on the interval (−∞, ∞) [8], i.e.,*${Ʈ}_{k}^{𝓎}\left({Ʈ}_{ᶇ}^{𝓎}\left({𝓍}_{1}\right)\right)={Ʈ}_{ᶇ}^{𝓎}\left({Ʈ}_{k}^{𝓎}\left({𝓍}_{1}\right)\right)={Ʈ}_{kᶇ}^{𝓎}\left({𝓍}_{1}\right)$.

It is worth noting that we obtain the original instance from [43] when we use 𝓎→0.

At this time, we should mention that the DL and CCP assignments are roughly DHP.

## 4. Proposed IBE Transformation Model for PKC under Human-Centered IoT Environments

We will now show our novel concept for converting a conformable Chebyshev chaotic maps-based cryptosystem into an IBE scheme under human-centered IoT environments. Please pay close attention to our essential KGS, since this is where the actual difference is made. Conformable Chebyshev chaotic maps-based cryptosystems can be easily turned into IBE schemes by effectively articulating private keys.

### 4.1. Setup Phase

1. Private Key Generator (PKG) selects any к users who refuse to work together. The minimum bit size of the user’s identity is then determined by the security limitation. Now, let ʠ be a huge prime, s. t. ƥ|(ʠ−1) and let $G_{𝓎,𝓆}=\left\{y^{0},y^{1},\ldots \ldots .,y^{ƥ-1}\right\}\;$ be a subgroup of the multiplicative group $Z_{ʠ}^{*}$ with prime ƥ order , where y is an order ƥ prime generator, and 𝛾∈[0,1] is a random rational number. Suppose that 𝑣 and $u={Ʈ}_{v}^{𝛾}\left(y\right)\left(\;mod\;ʠ\right)\;$ and 𝑣 are the public key and secret key of PKG.
2. PKG chooses private info $\left\{{ʂ}_{1},\;{ʂ}_{2},\ldots ,\;{ʂ}_{к}\;\right\}\;$ at random, where $\overset{к}{\underset{i=1}{\sum }}{ʂ}_{i}<ƥ$ and the consistent public info $\left\{{ƥ}_{1},\;{ƥ}_{2},\;\ldots ,{ƥ}_{к}\right\}$, where ${ƥ}_{i}={Ʈ}_{{ʂ}_{i}}^{𝛾}\left(y\right)\left(\;mod\;ʠ\right),\;\forall i\in \left(1,к\right)$.
3. Each user U has a distinct к-bit identity $id_{Ʋ}=\left(id_{Ʋ1},\;id_{Ʋ2},\;\ldots ,\;id_{Ʋк}\right)$, where $id_{Ʋi}$ ∈{0, 1}, ∀i∈(1,к).
4. Express the hash function $һ:\;\left\{0,\;1\right\}\to Z_{ʠ}^{*}\;.$

### 4.2. Key Generation Phase

**Figure 2:**
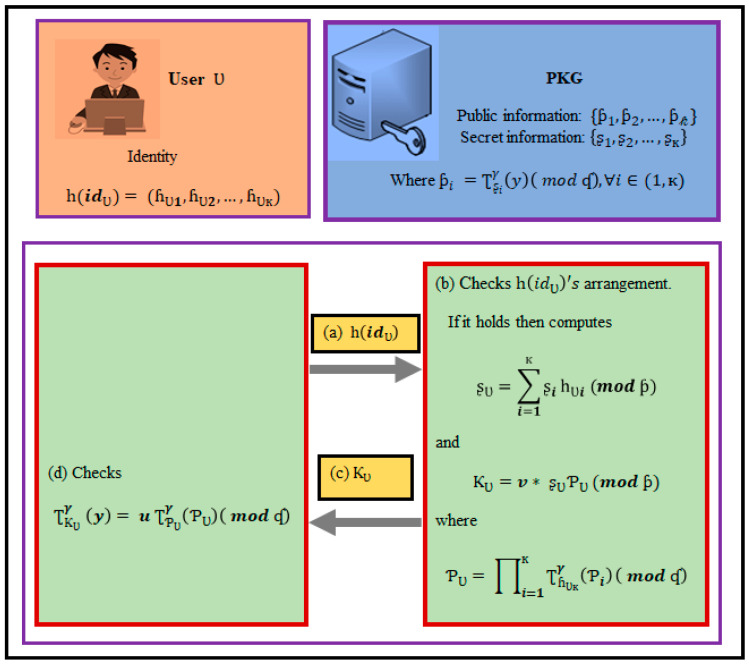
Private Key Generation (PKG) under the IoT environments.

Suppose that, for the sake of argument, that a user Ʋ wishes to begin the procedure. The private key is then generated using PKG and the key generation phase. The generation of the private key is depicted in Figure 2.

1. A user gives PKG her/his hashed identification $һ\left(id_{Ʋ}\right)=\left({ɦ}_{Ʋ1},\;{ɦ}_{Ʋ2},\;\ldots ,\;{ɦ}_{Ʋк}\right)$, where ${ɦ}_{Ʋi}$ $\in {𝕫}_{ʠ}^{*}$, ∀i∈(1,к).
2. PKG examines whether an identity $һ\left(id_{Ʋ}\right)\;$follows a given pattern. At that point, the identity is verified, PKG uses its secret information to compute ${ʂ}_{Ʋ}=\overset{к}{\underset{i=1}{\sum }}{ʂ}_{i}{һ}_{Ʋi}\;\left(mod\;ƥ\right)$.

(3) $${Ƙ}_{Ʋ}=v*\;{ʂ}_{Ʋ}{ƥ}_{Ʋ}\;\left(mod\;ƥ\right)$$

where ${ƥ}_{Ʋ}=\overset{к}{\underset{i=1}{\prod }}{Ʈ}_{{ɦ}_{Ʋк}}^{𝛾}\left({ƥ}_{i}\right)\left(\;mod\;ʠ\right)$.

3. PKG secretly transmits ${ᶄ}_{Ʋ}\;$to Ʋ as Ʋ’s private key.
4. Ʋ checks whether the condition ${Ʈ}_{{Ƙ}_{Ʋ}}^{𝛾}\;\left(y\right)=u\;{Ʈ}_{{ƥ}_{Ʋ}}^{𝛾}\left({ƥ}_{Ʋ}\right)\left(\;mod\;ʠ\right)$ holds, where ${ƥ}_{Ʋ}=\overset{к}{\underset{i=1}{\prod }}{Ʈ}_{{ɦ}_{Ʋк}}^{𝛾}\left({ƥ}_{i}\right)\left(\;mod\;ʠ\right)$ can be deduced from public data without any disagreement.

The accuracy of the given equation can be demonstrated in the following manner:

$${Ʈ}_{{Ƙ}_{Ʋ}}^{𝛾}\;\left(y\right)\left(\;mod\;ʠ\right)={Ʈ}_{\left(v*{ʂ}_{Ʋ}{ƥ}_{Ʋ}\right)}^{𝛾}\left(y\right)\left(\;mod\;ʠ\right)={Ʈ}_{v}^{𝛾}\left(y\right)*({Ʈ}_{{ƥ}_{Ʋ}}^{𝛾}\left(\xi_{{ʂ}_{Ʋ}}\left(𝓎\right)\right)\left(\;mod\;ʠ\right)\;=u\;{Ʈ}_{{ƥ}_{Ʋ}}^{𝛾}\left({ƥ}_{Ʋ}\right)\left(\;mod\;ʠ\right)$$

Note:$\;{ƥ}_{i}={Ʈ}_{{ʂ}_{i}}^{𝛾}\left(y\right)\left(\;mod\;ʠ\right),\;\forall i\in \left(1,к\right)$. (Setup phase 3.1 point 3)

### 4.3. IBE Transformation Model for PKC

A conformable Chebyshev chaotic maps-based cryptographic system can be easily converted to an ID-based cryptographic system using the key generation procedure under human-centered IoT environments. Given a large prime number ʠ such that ƥ|(ʠ−1) convoyed by a parameter $y\in Z_{ʠ}^{*}$, the conformable Chebyshev chaotic maps-based system (CCCMS) can be demarcated as $\mathrm{CCCMS}=\left\{\left(ʠ,y,\;v,\;u\right):u={Ʈ}_{v}^{𝛾}\left(y\right)\right\}$, where ʠ, y, u and v are public and secret keys, respectively. The projected ID-based encryption transformation procedure under human-centered IoT environments is described as follows:

*a*. *Describe the formation of the identity*

Users just use their identities as their public keys, which is the most important characteristic of an ID-based cryptography approach under human-centered IoT environments. As a result, the first step is to see if the identity corresponds to a preset formation.

*b*. Calculate the private key according to the instructions provided by the key generation (KG) process

A user Ʋ, for example, will get their public keys as a result of the key generating process. Consequently, in the proposed technique, both $\left\{{ʂ}_{1},\;{ʂ}_{2},\ldots ,\;{ʂ}_{к}\;\right\}\;$and u will be made public. Anyone can quickly determine the corresponding public assessment of Ʋ in such a design by computing:

$U_{Ʋ}={Ʈ}_{{ᶄ}_{Ʋ}}^{𝛾}\left(y\right)=u\;{Ʈ}_{{ƥ}_{Ʋ}}^{𝛾}\left({ƥ}_{Ʋ}\right)\left(\;mod\;ʠ\right)$, where

(4) $${ƥ}_{Ʋ}=\prod_{i=1}^{к}{Ʈ}_{{ɦ}_{Ʋк}}^{𝛾}\left({ƥ}_{i}\right)\left(\;mod\;ʠ\right)$$

As a result, the projected transformation procedure converts conformable Chebyshev chaotic maps-based cryptosystems into conformable Chebyshev chaotic maps-based ID-based encryption schemes, where ${Ԟ}_{Ʋ}\;$is preserved as a private key and $u_{Ʋ}$ is the consistent public key.

Our new model may convert any conformable Chebyshev chaotic maps-based cryptosystem into an ID-based encryption method because the user’s identity is the only key involved in the transformation procedure.

### 4.4. Verification of the Transformation Mechanism

We show how our new mechanism works under the human-centered IoT environments in this segment. Now, let us iterate that our signature system is built on conformable Chebyshev chaotic maps. Let m be the text Ʋ want to sign, $v_{Ʋ}$ be the private key of Ʋ, and $u_{Ʋ}={Ʈ}_{v_{Ʋ}}^{𝛾}\left(y\right)\left(mod\;ʠ\right)$ be the corresponding public key of Ʋ. The signature system based on conformable Chebyshev chaotic maps can be formulated as: with the key pair (KP) $\left\{\left(ʠ,\;y,\;v_{Ʋ},\;u_{Ʋ}\right)\;:\;u_{Ʋ}={Ʈ}_{v_{Ʋ}}^{𝛾}\left(y\right)\left(mod\;ʠ\right)\right\}$ and an arbitrary private integer $r\in Z_{ƥ}^{*}$, and 𝛾∈[0,1] as a random rational number.

$Sig_{KP}\left(m,r\right)=\left(ⱳ,\;ɓ\right)$, where $ⱳ={Ʈ}_{r}^{𝛾}\left(y\right)\left(mod\;ʠ\right)\;$ and $ɓ=\left(\frac{m}{rv_{Ʋ}ⱳ}\right)\;\left(mod\;ƥ\right)$

The verification is formulated as follows for $m,\;ⱳ\in Z_{ʠ}^{*}$ and $ɓ\in Z_{ƥ}^{*}$:

$$Ver_{KP}\left(m,ⱳ,\;ɓ\right)=true\;\Leftrightarrow {Ʈ}_{m}^{𝛾}\left(y\right)={Ʈ}_{ɓ}^{𝛾}\left(ⱳ\right)\;{Ʈ}_{ⱳ}^{𝛾}\left(u_{Ʋ}\right)\;\left(mod\;ʠ\right)$$

The accuracy of the preceding equation can be verified as follows:

$${Ʈ}_{ɓ}^{𝛾}\left(ⱳ\right)\;{Ʈ}_{ⱳ}^{𝛾}\left(u_{Ʋ}\right)\;\left(mod\;ʠ\right)={Ʈ}_{ɓ}^{𝛾}\left({Ʈ}_{r}^{𝛾}\left(y\right)\right){Ʈ}_{ⱳ}^{𝛾}\left({Ʈ}_{v_{Ʋ}}^{𝛾}\left(y\right)\right)\left(mod\;ʠ\right)={Ʈ}_{ɓr}^{𝛾}\left(y\right){Ʈ}_{ⱳv_{Ʋ}}^{𝛾}\left(y\right)\left(mod\;ʠ\right)\;={Ʈ}_{m*{\left(v_{Ʋ}ⱳ\right)}^{-1}}^{𝛾}\left(y\right){Ʈ}_{ⱳv_{Ʋ}}^{𝛾}\left(y\right)\left(mod\;ʠ\right)={Ʈ}_{m}^{𝛾}\left(y\right)$$

To summarize our approach, we use conformable Chebyshev chaotic maps to create a novel ID-based signature scheme:

1. Describe the identity prearrangement for Ʋ as $һ\left(id_{Ʋ}\right)$.
2. For example, during the key generation step, Ʋ will receive their secret value. Now $KP=\{\left(ʠ,\;y,\;v_{Ʋ},\;u_{Ʋ}\right)\;:\;u_{Ʋ}={Ʈ}_{v_{Ʋ}}^{\gamma }\left(y\right)\left(mod\;ʠ\right)\;$ is translated into an ID-based encryption model as $IDKP=\left\{\left(ʠ,\;y,{ᶄ}_{Ʋ},U_{Ʋ}\;\right):\;U_{Ʋ}={Ʈ}_{{ᶄ}_{Ʋ}}^{\gamma }\left(y\right)\left(mod\;ʠ\right)\;\right\}$, where ${ᶄ}_{Ʋ}$ is determined by Equation (3), and $U_{Ʋ}$ is determined by Equation (4). In these lines, the original signature structure based on conformable Chebyshev chaotic maps can be rewritten as $Sig_{KP}\left(m,r\right)=\left(ⱳ,\;ɓ\right)$, where $ⱳ={Ʈ}_{r}^{𝛾}\left(y\right)\left(mod\;ʠ\right)\;$ and $ɓ=\left(\frac{m}{rv_{Ʋ}ⱳ}\right)\;\left(mod\;ƥ\right)$.

The verification is described as follows for a given $m,\;ⱳ\in Z_{ʠ}^{*}$ and $ɓ\in Z_{ƥ}^{*}$:

$$Ver_{KP}\left(m,ⱳ,\;ɓ\right)=true\;\Leftrightarrow {Ʈ}_{m}^{𝛾}\left(y\right)={Ʈ}_{ɓ}^{𝛾}\left(ⱳ\right){Ʈ}_{ⱳ}^{𝛾}\left(U_{Ʋ}\right)\;\left(mod\;ʠ\right)$$

By employing conformable Chebyshev chaotic maps, we can surely implant the logic of ID-based cryptography into novel signature approaches, such as the ElGamal signature [54] and discrete log-based signature procedures [55].

## 5. Security Examination and Performance Investigation

In this section, we present the security examination and performance investigation of the proposed IBE transformation model. In particular, the security examination is given in Section 5.1, and the performance investigation is given in Section 5.2.

### 5.1. Security Examination

**Figure 3:**
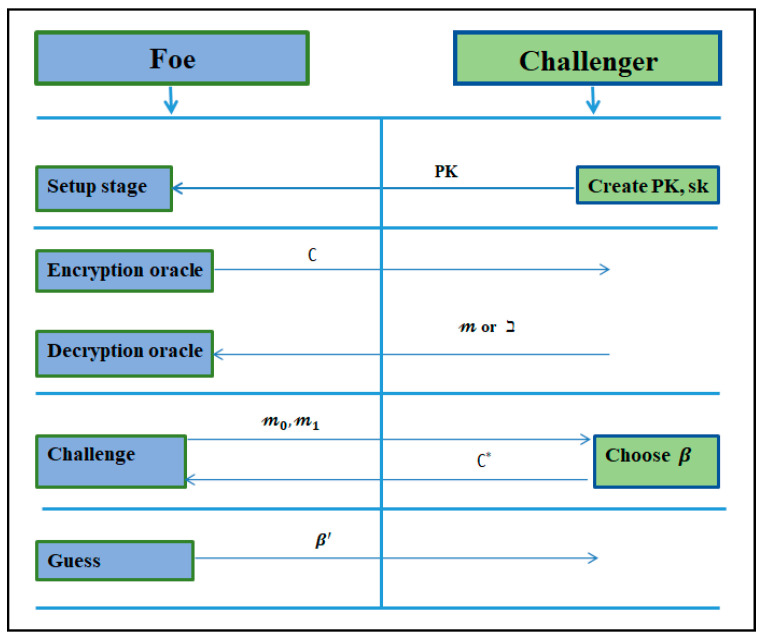
Illustrative representation of IND-CCA.

The chosen-ciphertext attack (IND-CCA) [56,57] is a typical security test against which a public key cryptography system must be tested. By proposing IND-ID-CCA, Boneh and Franklin [16] strengthened chosen-ciphertext security for the IBC techniques, where a Ƒ gets to choose an objective public key to attack adaptively, even if it is not the challenger’s overall identity. Certainly, IND-ID-CCA is currently the most stringent security necessity on an IBC system, as it provides the adversary with the greatest ease and capability to attack. Canetti et al. [61] then described another security concept for IBC systems, in which the adversary must send an initial signal indicating that it will attack. This type of attack is known as IND-sID-CCA. IND-CCA and IND-sID-CCA are now specified as follows:

**Definition 4.**  *A PKC procedure is said to be IND-CCA secure if*∃ *no probabilistic polynomial time (PPT), foe*Ƒ*has a non-negligible advantage [56,57], as shown in Figure 3*.

**Figure 4:**
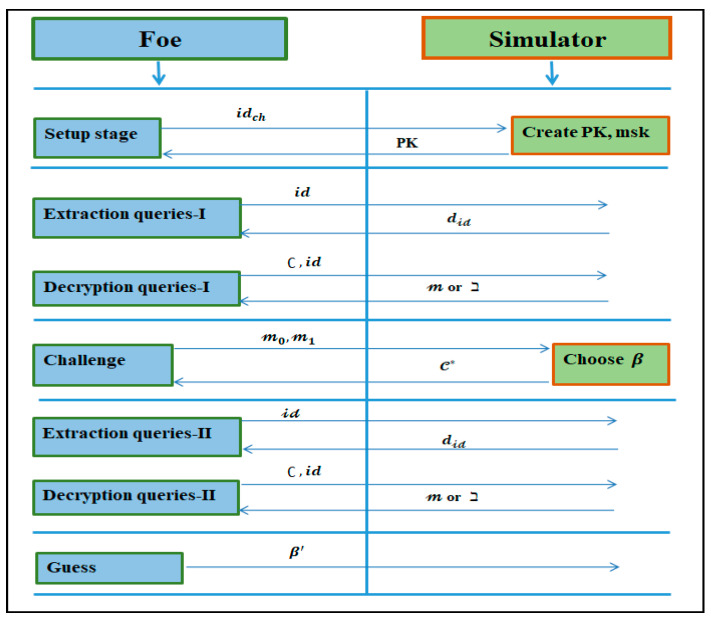
Illustrative representation of IND-sID-CCA.

**Definition 5.**  *An IBE procedure*ℐ*is said to be IND-sID-CCA secure if*∃ *no PPT foe*Ƒ*has a non-negligible advantage [61], as demonstrated in Figure 4*.

Now, let us examine the security of the proposed new approach. Our goal is to carry out the following tests: (1) To demonstrate that the presented transformation procedure is accomplished by interpreting a well-designed and secure conformable Chebyshev chaotic maps-based scheme into an equally robust IBE under the human-centered IoT environments. (2) To test the ID-based system against IND-sID-CCA proposed by Canetti et al. [61] in the ROM, we employed a reductionist method. On the assumption that the inputted PKC employing conformable Chebyshev chaotic maps is IND-CCA secure, the findings showed that our suggested approach is IND-sID-CCA secure.

**Theorem 1.**  *Let*ɦ*be a random oracle. If the original conformable Chebyshev chaotic maps cryptosystem is IND-CCA secure, the recommended ID-based cryptosystem employing conformable Chebyshev chaotic maps is IND-sID-CCA secure. Assume an IND-sID-CCA foe Ƒ with advantage ϵ(*к*), which opposes the ID-based cryptosystem based on conformable Chebyshev chaotic maps. Then, in contrast to the cryptosystem utilizing conformable Chebyshev chaotic maps, there exists an IND-CCA adversary with an advantage of at least*ϵ(к)*. It has an*O(*time*(F)) *execution time*.

**Proof.**  In an IND-CCA game, the basic premise of the validation is to create an IND-CCA foe Ƒ to get an advantage over the PKC utilizing conformable Chebyshev chaotic maps. □

The IND-CCA challenger constructs PK=〈ʠ,y, u〉 and SK v that satisfies $u={Ʈ}_{v}^{𝛾}\left(y\right)\left(mod\;ʠ\right)$. The challenger gives PK to the Ƒ, then launches an IND-CCA attack with the help of Ƒ as follows:

Initialization phase: The Ƒ produces an identity $id_{ch}\;$ that it wishes to be contested.

Setup phase: The challenger begins the method of setup. The scheme parameters have now been supplied to the foe. It protects itself by keeping the master key.

һ-queries: In order to respond to an һ-inquiry, it is appropriate to maintain a list of tuples $\;\langle id_{Ƒi},U_{Ƒi},{ᶄ}_{Ƒi}\rangle$, which we refer to as a list $L_{һ}$. The list is empty at the start. When Ƒ asks for $id_{Ƒi}$ at a specific moment, it replies as follows: If the enquiry appears on ${ℒ}_{һ}$ in the tuple $\;\langle id_{Ƒi},U_{Ƒi},{ᶄ}_{Ƒi}\rangle$, then reacts with $һ\left(id_{Ƒi}\right)=u_{Ƒi}$.

1. Else, if $\langle id_{Ƒi}\neq id_{ch}\rangle \;$is true, it generates an arbitrary ${ᶄ}_{Ƒi}\in Z_{ʠ}^{*}$ and processes $U_{Ƒi}={Ʈ}_{{ᶄ}_{Ƒi}}^{𝛾}\left(y\right)\left(mod\;ʠ\right)$, otherwise Ƒ sets ${ᶄ}_{Ƒi}=\mu$ and $U_{Ƒi}=u$. mu is a special notation in this case.
2. Ƒ adds the tuple $\langle id_{Ƒi},U_{Ƒi},{ᶄ}_{Ƒi}\rangle$ to $L_{һ}\;$and returns $U_{Ƒi}$ to Ƒ.

Step 1 extraction queries: When Ƒ asks for the private key for $id_{Ƒi}$, it calls the above process, which returns $һ\left(id_{Ƒi}\right)=u_{Ƒi}$, where$\;\langle id_{Ƒi},U_{Ƒi},{ᶄ}_{Ƒi}\rangle$ is the equivalent entry in $L_{һ}$. Because $U_{Ƒi}={Ʈ}_{{ᶄ}_{Ƒi}}^{𝛾}\left(y\right)\left(mod\;ʠ\right)$, the genuine private key ${ᶄ}_{Ƒi}$ for $id_{Ƒi}$ maybe recovered. The $id_{ch}$ extraction enquiry will be dismissed.

Step 1 decryption queries: Let ∁ be the ciphertext of the conformable Chebyshev chaotic maps-based PKC, and $\langle id_{Ƒi},\;{∁}_{i}\rangle$ be a decryption investigation supplied by Ƒ. Ƒ responds to the question in the following way:

1. If $\langle id_{Ƒi}\neq id_{ch}\rangle$ is true. Then the һ-inquiry method is executed to make $\langle id_{Ƒi},U_{Ƒi},{ᶄ}_{Ƒi}\rangle$ the connecting tuple on ${ℒ}_{һ}$. Then it uses ${ᶄ}_{Ƒi}$ to respond to the decryption question.
2. If $\langle id_{Ƒi}=id_{ch}\rangle$ is true, and the decryption inquiry is executed by Ƒ with $\langle {∁}_{i}\rangle$ and the response of the challenger are transferred back to Ƒ.

Challenge: When Ƒ determines that Phase 1 is complete, it returns$\;m_{1},\;m_{0}\in \left(-\infty ,+\infty \right)$, which it wishes to be challenged on. After that, Ƒ reacts as follows:

1. The challenger receives $\;m_{1}$ and $m_{0}$ from Ƒ. The challenger responds to the PKC’s ∁ s. t. ∁ is the encryption of $m_{\beta }\;$for any coin β∈{0, 1}.
2. Ƒ executes the һ-query method to retrieve $u\in Z_{ʠ}^{*}$ so that $һ\left(id_{ch}\right)=u$ and responds with a ∁ to Ƒ.

Stage 2 extraction inquiries: Except for the extraction query on$\;id_{ch}$, which will be refused, reacts in the same way as in Stage 1.

Stage 2 decryption inquiries: Except for the decryption query$\;\langle id_{Ƒi},∁\rangle$, which will be rejected, Ƒ reacts similarly to Stage 1.

Guess: Ƒ eventually offers an β’ guess for β. As a guess for β, foe Ƒ comes up with β’.

The responses to һ-inquiries are identical to what will occur in real-world attacks. In the meantime, in $Z_{ʠ}^{*}$, every response is uniformly and freely spread. The full responses to decryption and SK extraction queries are valid. Thus, the Ƒ will not abort for the simulation period; particularly, the probability of flawless simulation is 1. Following this, we can assume the foe Ƒ has fruitfully played the adversary and hurled a true attack. We obtained the result |Pr[β=β’]−1/2| ≥ϵ(к), through the explanation of method Ƒ, which at least has advantage ϵ(к) over the PKC utilizing conformable Chebyshev chaotic maps. This concludes the proof and verifies hypothesis 1.

### 5.2. Performance Investigation 

To highlight the efficacy of our original design, we compare our new IBE transformation model to four earlier strategies presented by [62,63,64,65]. Our evaluation findings are presented using the notations ${⟙}_{exp},\;{⟙}_{chaos},{⟙}_{inv\;},\;{⟙}_{mul\;}$ and ${⟙}_{hash}$. We depict the execution time for a group modular exponentiation $\;\left({⟙}_{exp}\right)$, a chaotic map operation$\left(\;{⟙}_{chaos}\right)$, one modular inverse operation $\left({⟙}_{inv\;}\right)$, a modular multiplication $\left({⟙}_{mul\;}\right)$, and a one-way hash function $\left({⟙}_{hash}\right)$ in the decryption and encryption stages. It is worth noting that only the encryption and decryption processes require more processing power than the setup and key generation stages. We examine the stages by comparing the computational expenses of our current IBE transformation model to the works of [62,63,64,65].

**Figure 5:**
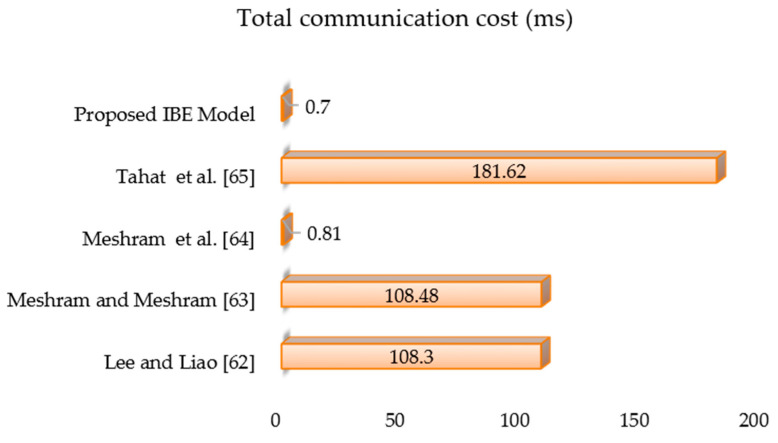
Total communication cost (ms) analysis of the proposed model with other models.

Table 3 lists the functions of the proposed IBE transformation model, and Figure 5 compares the computational costs of relevant models by [62,63,64,65]. Based on the findings of the tests in [6,8,60,66] we arrive at the following computation time statistics with unit hashing time: ${⟙}_{exp}=600{⟙}_{hash}$, ${⟙}_{mul\;}=2.5{⟙}_{hash}\;,\;{⟙}_{inv\;}=7.5{⟙}_{hash}$ and ${⟙}_{hash}\approx {⟙}_{chaos}$. The order of computational complexity in this method is as follows: ${⟙}_{hash}\approx {⟙}_{chaos}<{⟙}_{mul\;}<{⟙}_{inv\;}<{⟙}_{exp}$. The computation time of the cryptographic primitives was measured using a 32-bit Cortex-M3 microcontroller running at 72 MHz in a simulation hardware environment [67]. A one-way hash function takes 0.06 milliseconds (ms) [8,67] and that [𝛾=0.5] [8]. Total communication costs for references by [62,63,64,65], as well as the proposed IBE transformation model, are 108.3 ms, 108.48 ms, 0.81 ms, 181.62 ms, and 0.70 ms, respectively. It should be noted that the transformation model based on extended conformable Chebyshev chaotic maps created in this paper has a lower computing cost than [62,63,64,65] and is probably secure in a random oracle than [62,65]. The work of [64] is based on an extended chaotic map, but the proposed work is based on conformable Chebyshev chaotic maps. In our proposed IBE transformation scheme, the design leverages Chebyshev polynomials and conformable calculus. In particular, conformable calculus helps to significantly increase the security of the cryptographic scheme compared to the cryptographic scheme, which is based on chaotic maps, owing to the selection of arbitrary rational number 𝛾∈[0,1]. As a result, an adversary cannot calculate the arbitrary rational number to break the security of the proposed technique. Also, the proposed scheme requires minimum communication cost when compared to the scheme reported in [64]. We arrive at the following computation time values with unit hashing time based on the experimental results in [6,8,60,66].

## 6. Conclusions

This article has demonstrated how to build a provably secure IBE transformation model for PKC under human-centered IoT environments. The proposed model is designed based on a conformable Chebyshev chaotic map without changing the original PKC configuration. We chose conformable Chebyshev chaotic maps to achieve IBE transformation in avoiding the complexity and cost of inventing an entirely new IBE technique. Specifically, we demonstrated that our new model is secure in the ROM under the IND-sID-CCA. At a relatively low computing cost, this configuration may be easily transmitted to an existing system. Our new model, which combines the strengths of conformable Chebyshev chaotic maps and the IBE, is robust, secure, and poses broad application prospects. Our future work would design and develop a secure identity-based short signature transformation model for a short signature scheme under human-centered IoT environments.

## Figures and Tables

**Table 1:** Comparison of related works with our proposed work.

Ref.	Related Works	Limitations of Related Works	Our Proposed Scheme
Boneh and Franklin [15,16]	Identity-based encryption from the Weil pairing was projected.	The scheme has a high computation processing time.	Our proposed IBE transformation model provides an extremely low computational processing time.
Sakai–Kasahara [17]	A bilinear pairing-based IBE scheme, which is faster than the Boneh and Franklin scheme, was reported.	The scheme possesses computationally intensive characteristics, which limits its usefulness in lightweight IoT-centered environments.	The projected IBE transformation model supports resource-constrained lightweight devices in IoT and human-centered environments.
Liu et al. [21]	An efficient, provably secure IBS technique that uses multi-time usage of offline storage was proposed.	It is seen to take a long time for signing a few messages. This poses a significant limitation, especially when it is applied in complex wireless networks.	Our new model provides the desired offline storage and client-friendliness compared to the IBS technique that uses multi-time usage of offline storage.
Liu and Zhou [39]	An efficient IBE scheme with short ciphertexts was presented.	The model can be executed ‘offline’ or inside some powerful devices only. This limits its usefulness in extremely lightweight devices and applications.	Our new procedure is accomplished by interpreting a well-designed and secure conformable Chebyshev chaotic maps-based scheme into an equally robust ID-based cryptosystem under human-centered IoT environments.
Lai et al. [42]	An ordinary IBE scheme was converted to an online/offline IBE scheme.	The method adopted to separate the computation of the receiver’s identity into offline and online phases is cumbersome, and the security is limited.	Our new model is secure in the ROM under the IND-sID-CCA.
Xu, Wu, and Xie [43]	An IBE scheme for lightweight devices was reported.	The model requires powerful devices to process heavy computations in the offline encryption phase. Additionally, the model is based on bilinear pairing on elliptic curves and requires point multiplication.	The proposed model is designed based on a conformable Chebyshev chaotic map without changing the original PKC configuration.
Pourasad, Ranjbarzadeh, and Mardani [46]	The work presents a novel method for digital image encryption leveraging chaos theory.	The scheme requires an extensive database for the digital images.	Our projected architecture showed that there is no need for an extensive public key database.
Parida et al. [47]	The work presents elliptic curve-based image encryption and authentication model that uses a secure elliptic curve Diffie–Hellman key exchange to compute a shared session key with the enhanced ElGamal encoding scheme.	The model uses the secure Elliptic Curve Diffie–Hellman(ECDH) key exchange to compute a shared session key along with the improved ElGamal encoding scheme, resulting in point multiplication operations, which are computationally expensive.	At a relatively low computing cost, our configuration may be easily transmitted to an existing system.
Pourjabbar Kari [48]	A new image encryption scheme based on hybrid chaotic maps was proposed.	The work extends the original grayscale image matrix to the square matrix by adding the sequences generated with proper chaotic maps to implement the first step of the diffusion phase. This procedure takes time and requires massive computational resources.	Our new model, which combines the strengths of conformable Chebyshev chaotic maps and the IBE, is robust, secure, and poses broad application prospects.
Talhaoui and Wang [49]	The work proposes a new fractional one-dimensional chaotic map with a sizeable chaotic space.	A new fractional one-dimensional chaotic map with a large chaotic space was employed, resulting in a longer processing time and huge communication costs.	The proposed work is based on conformable Chebyshev chaotic maps. The development of the IBE scheme depends on Chebyshev polynomial and conformable calculus, which facilitates low communication costs.

**Table 2:** List of mathematical symbols and their meanings.

Symbol	Meaning
${Ʈ}^{𝓎}$	Conformable Chebyshev chaotic maps
ʠ	Large prime number of bit length
ƥ	Large prime factors of ʠ−1
$id_{Ʋ}$	Identity of Ʋ user
𝛾	An arbitrary rational number
u	Public key
𝑣	Private key
һ	Hash function
r	Random number
m	Message

**Table 3:** Computational cost assessment of proposed IBE transformation model with other models.

Model	$F_{1}$	$F_{2}$	$F_{3}$
Lee and Liao [62]	$2{⟙}_{mul\;}+3{⟙}_{exp}$	𝓝	𝓝
Meshram and Meshram [63]	$2{⟙}_{mul\;}+3{⟙}_{exp}+3{⟙}_{hash}$	**Ƴ**	**Ƴ**
Meshram et al. [64]	$3({⟙}_{hash}+{⟙}_{chaos}+{⟙}_{mul\;})$	**Ƴ**	**Ƴ**
Tahat et al. [65]	$3{⟙}_{hash}+{⟙}_{inv\;}+5{⟙}_{exp}+4{⟙}_{chaos}+2{⟙}_{mul\;}$	𝓝	𝓝
Proposed IBE Model	${⟙}_{hash}+2{⟙}_{chaos}+{⟙}_{mul\;}$	**Ƴ**	**Ƴ**

Note: **Ƴ:** The model can withstand the danger, and 𝓝: The model cannot withstand the danger. $F_{1}$: Computational cost for framework performance (decryption and encryption); $F_{2}$: Provides provable security in the ROM; $\;\mathrm{and}\;F_{3}$: Provides security in CCA

## Data Availability

Data sharing is not applicable to this article.
